# Inattention, Working Memory, and Goal Neglect in a Community Sample

**DOI:** 10.3389/fpsyg.2016.01428

**Published:** 2016-09-22

**Authors:** Rebecca N. Elisa, Emili Balaguer-Ballester, Benjamin A. Parris

**Affiliations:** Department of Psychology, Faculty of Science and Technology, Bournemouth UniversityPoole, UK

**Keywords:** inattention, working memory, goal neglect, ADHD, executive function

## Abstract

Executive function deficits have been linked to attention deficit hyperactivity disorder (ADHD), but it has been theorized that the symptom inattention is specifically related to problems with complex verbal working memory (WM). Using the Conners Adult ADHD rating scale, adults aged 18–35 were assessed for ADHD symptoms, and completed tasks designed to tap verbal and spatial aspects of WM (Experiment 1). Results showed that high inattention predicted poor performance on both simple and complex verbal WM measures. Results relating to spatial WM were inconclusive. In a follow up experiment based on the theory that those with inattention have problems receiving verbal instructions, a measure of goal neglect assessing integration of information into a task model in WM was employed (Experiment 2). Results showed that high inattention uniquely predicted performance on this task, representing the first reported association between inattention and the phenomenon of goal neglect. The results from both experiments lend support to the WM theory of inattention.

## Introduction

Inattention is one of three core symptoms characterizing attention deficit hyperactivity disorder (ADHD); a childhood onset, neurodevelopmental disorder. It is characterized by an inability to focus, high levels of distractibility, forgetfulness, and poor organization and planning. The two other symptoms associated with ADHD, hyperactivity and impulsivity, are characterized by excessive energy levels, impatience, and disruptive, often inappropriate behavior, with a lack of regard for social rules. The Diagnostic and Statistical Manual of Mental Disorders 5th edition (American Psychiatric Association, 2013) lists three presentations of ADHD; the combined type where individuals meet criteria for both inattention and hyperactivity-impulsivity (ADHD-C), the predominantly inattentive type (PIT) where individuals only meet criteria for inattention (ADHD-I), and the predominantly hyperactive-impulsive type (ADHD-HI) where only hyperactive-impulsive criteria are met.

Most of the existing work on this disorder focuses on participants with combined symptoms, and some of the work makes no reference to subtype at all. It is not possible to draw conclusions specifically about inattention in these cases. This is important, as several authors have argued that ADHD-I is likely qualitatively different from ADHD-C, and should perhaps be considered a disorder in its own right with distinct etiology, symptoms, comorbidities, and cognitive profile (Barkley, 1997, 2001; Milich et al., 2001; Diamond, 2005). Furthermore, describing pure inattention as ADHD-I is something of a misnomer because of the implied hyperactivity. Therefore, in this work diagnosis of the PIT will be referred to as such in order to avoid confusion (as in Barkley, 2001). Furthermore, although most commonly associated with ADHD, inattention is not specific to it, and is also a symptom of other disorders such as autism spectrum disorder (Mayes et al., 2012) schizophrenia (Egeland et al., 2003), and some eating disorders (Seitz et al., 2013), warranting further investigation of this symptom/disorder as an independent entity.

### Inattention in Adulthood

For many years ADHD was thought to be a disorder exclusive to childhood, and has only recently been recognized as existing in adults. The latest version of the DSM, released in 2014, was the first to provide specific diagnostic criteria for adults. Longitudinal follow-up studies suggest that ADHD persists into adulthood in around 60% of cases (Elliott, 2002), and that up to 6% of adults may have ADHD (Wender et al., 2001). These figures could be even higher when we consider that the diagnostic criteria developed for children has been deemed by many as unsuitable for application to adult populations, and may have resulted in under-diagnosis (Murphy and Barkley, 1996). It is difficult to generalize findings from child research to adults, as the disorder’s manifestation appears to differ. Research suggests that symptoms of hyperactivity and impulsivity diminish with age, while inattention persists (Biederman et al., 2000). It is therefore not surprising that the majority of adults diagnosed with ADHD present with PIT (Millstein et al., 1997); the most common complaints being cognitive (e.g., difficulty concentrating, forgetfulness), and self-regulatory (e.g., problems with organization and planning, poor discipline), none implying hyperactivity (Wolf and Wasserstein, 2001).

### Inattention in the General Population

Recent research and subsequent debate has led many experts to a shift in approach, from a categorical, to a dimensional view of ADHD symptoms (Barkley and Murphy, 2006). It is suggested that they are better regarded as being at the extreme end of normal expression within the general population. This is supported by the prevalence of symptoms in community samples (Faraone and Biederman, 2005; Alloway et al., 2010). With this in mind, the aim of this research is to explore inattention as a symptom in its own right, as it appears within the general population, rather than one as part of a disorder. As the majority of work on inattention falls within the ADHD literature, this is necessarily the main frame of reference. However, inattention is the primary focus of this work.

### Inattention and Working Memory

Much of the research on ADHD has focused on neuropsychological deficits. This literature has established a reliable link between ADHD and executive functions (EFs) in children (Doyle, 2005; Willcutt et al., 2005), and in adults both clinically, and in community samples, and EF’s (cf. Johnson et al., 2001; Woods et al., 2002; Hervey et al., 2004; Boonstra et al., 2005; Schoechlin and Engel, 2005; Alderson et al., 2013). However, this research is largely relevant only to those with hyperactivity-impulsivity (HI). Indeed, Barkley’s (1997) influential EF model of ADHD is intended to describe only those with ADHD-C, not PIT. There is however, reason to believe that there may be differences in neuropsychological profile between subtypes (Chhabildas et al., 2001; Nigg et al., 2002; Schmitz et al., 2002). Diamond (2005) posits that the defining EF impairment in PIT is in working memory (WM), and that deficits in this particular EF are associated with inattention alone; not hyperactivity or impulsivity. She suggests that *complex-span tasks*, i.e., those that require working with information under high interference conditions, will be most sensitive to the WM problems experienced with inattention.

Working memory has been defined in several ways. All agree that it has a limited capacity, and requires holding information in mind in an active, easily retrievable form, and that it is distinct from short-term memory. Baddeley and Hitch (1974) defined WM as holding information in mind combined with some kind of ongoing mental activity. This can mean manipulating the information being held, or performing an entirely separate but simultaneous operation. Another model of WM defines it as the ability to hold information in mind whilst blocking or inhibiting counter-productive information (Conway and Engle, 1994; Kane and Engle, 2000, 2002). The WM model of Baddeley and Hitch has three components; the central executive, the visuospatial sketchpad and the phonological loop. The central executive is responsible for coordinating attention, and has the use of two “slave systems” for the storage of verbal (phonological loop) and visual/spatial (visuospatial sketchpad) information. Moreover, Baddeley (2000) proposed a further slave system called the *Episodic Buffer* whose role was to integrate phonological, visual and spatial information and has recently been associated with the phenomenon of goal neglect (Duncan et al., 1996). In many ways the central executive of WM, and attention, are overlapping constructs; indeed it is sometimes referred to as executive attention. Deficits in WM associated with inattention are therefore thought to be related to this component of the model.

The literature on WM and inattention in children in both clinical and community samples tends to support Diamond’s view (Klingberg et al., 2005; Martinussen and Tannock, 2006; Lui and Tannock, 2007). A study that looked at differences in WM performance between the subtypes ADHD-C and PIT in adults, found only weak evidence that PIT may be related to greater impairment (Schweitzer et al., 2006). However, without an ADHD-HI group to compare to, it is difficult to ascertain the role of inattention in any impairment, as both groups performed significantly poorer than normal controls. A study comparing ADHD-HI and PIT groups of adults found only the participants with PIT had a deficit in WM compared to controls, however, group sizes were notably small (Gansler et al., 1998). In a non-clinical sample of adults, Kim (2004) found inattention was predicted by verbal WM performance. Other research on non-clinical inattention in adults has found inattention to be the only symptom of ADHD to predict performance on a reasoning task strongly correlated with WM (the Cognitive Reflection Test; Stupple et al., 2013; Elisa and Parris, 2015).

There is also the question of whether the impairment is with a specific type of WM. Diamond writes that verbal presentation of material places a particularly high demand on WM, and that children with PIT often have superior spatial skills. This would suggest that the key problem may be with verbal WM. However, research testing combined-type or ADHD samples of children and adults without mention of subtypes has produced mixed findings. Several studies have found an impairment in spatial WM (Dowson et al., 2004; Westerberg et al., 2004). Others have found specific impairments in verbal WM (Seidman et al., 1998; Marchetta et al., 2008). Two meta-analyses found impairments across both types of WM (McInnes et al., 2003; Martinussen et al., 2005; Kasper et al., 2012). In a non-clinical sample of children, Lui and Tannock (2007) found composite WM including verbal and spatial measures predicted parent-rated inattention (but not hyperactivity-impulsivity). The mixed findings and lack of consideration for ADHD subtypes in some of these studies makes them difficult to interpret for present purposes.

The present research was designed to test Diamond’s hypothesis that inattention, but not hyperactivity or impulsivity, would be associated with impairments in performance on a complex, but not a simple, verbal WM task. Given the putative dimensional nature of inattention and other ADHD symptoms, we tested a community sample of adults. We are not aware of any prior research that has looked at complex vs. simple WM tasks in relation to either clinical or non-clinical ADHD symptoms. Furthermore, we employed a variety of tests of WM capacity. Experiment 1 employed traditionally used measures of WM capacity, specifically the Backward Digit Span task and the Operation Span (OSPAN) task (Turner and Engle, 1989) to represent both simple and complex-span varieties, respectively. We predicted that inattention but not hyperactivity or impulsivity would explain unique variance on the OSPAN task, but that a relationship may not be evident between inattention and performance on the backward digit span task. Although, the literature does not lead to a strong prediction, in line with Diamond’s point on the importance of verbally presented material, we expected that inattention may not explain variance in a spatial WM task. The Corsi blocks task (Corsi, 1973) was used to assess spatial WM span.

## Experiment 1

### Method

#### Participants

Ninety-five males (*N* = 30) and females (*N* = 65) aged 18–35 years (*M* = 21.46, *SD* = 4.19) were recruited for this research, largely through opportunity sampling. The majority of participants were psychology students from Bournemouth University, who collected course credits for their time. None of the participants had an existing ADHD diagnosis. All participants gave written informed consent to participate in the research, which was approved by Bournemouth University Ethics Committee.

#### Materials

##### ADHD symptoms

*Conners Adult ADHD Rating Scale–Self-Report: Short Version (CAARS-S:S;* Conners et al., 1999). This is a 26 item self-report measure designed to assess current ADHD symptoms in adults. Items are rated on a four-point Likert-type scale, where 0 = *not at all* and 3 = *very much.* The measure contains four factor-derived subscales; (A) inattention/memory problems, (B) hyperactivity/restlessness, (C) impulsivity/emotional lability, (D) problems with self-concept, as well as (E) an ADHD index comprised of items from the other subscales. For each subscale, a *T*-score is derived. Guidelines suggest that a *T*-score of 45–55 is average for adults (using data from a normative sample). Scores range from 29 to 90. A *T*-score of above 65 is considered to be indicative of clinically elevated symptoms.

##### Working memory span tasks

*Backward Digit Span (BDS).* This is a test of verbal WM, and requires participants to maintain information online while mentally manipulating that same information. Participants were presented with series of digits spoken verbally by the experimenter. After presentation of each series, participants were instructed to verbally repeat the numbers back to the experimenter in the opposite order to presentation. Series consisted of 2–8 digits with two trials for each length. Testing stopped after both items of a trial were failed or all trials were completed. One point was awarded for each correct trial, giving a maximum possible score of 14.

*Operation Span (OSPAN;* Turner and Engle, 1989). This task also tests verbal WM, and requires participants to hold information online while intermittently processing unrelated information. Participants were shown a series of operation-word strings (ranging from two to six in length) presented on a computer. These consisted of simultaneously presented mathematical equations, and unrelated words to be recalled, for example:

(9/3)⁢+ 2=5⁢? Beach

Participants were instructed to read the equation and indicate by key press whether the answer presented was correct or not. They were told to then read the word aloud. This continued until the end of the set at which point participants were asked to recall and type in all the words from that set. Three sets of each length were presented, and appeared in an unpredictable order so that the number of words to recall was unknown until recall.

A partial-credit unit scoring (where each item is scored as a proportion of correctly recalled elements per item, regardless of item size) method was used for this test as recommended by Conway et al. (2005) and Redick and Lindsey (2013). This gave scores that ranged from 0 to 100.

*Corsi Blocks* (Corsi, 1973). This version of the traditional measure of visuospatial WM was taken from the Psychology Experiment Building Language (PEBL) battery of tests. Participants viewed a series of blocks lighting up on screen and were required to reproduce the order they were lit in by mouse click on the correct blocks. Series were 2–8 blocks in length with two trials for each length. Testing stopped after both items of a trial were failed or all trials were completed. One point was awarded for each correct trial, giving a maximum possible score of 14.

##### Intelligence quotient (IQ)

A shortened version of the Wechsler Abbreviated Scale of Intelligence (WASI-II) was administered. This consisted of vocabulary and matrix reasoning subsets so that a score for both crystallized (Gc) and fluid intelligence (Gf) was obtained. An approximate overall WAIS IQ score was also calculated for each participant. IQ is a known correlate of WM, and was included as a measure in order to control for covariance.

### Procedure

Each participant was individually administered each test item alone, in a quiet testing room. Test administration order was counterbalanced.

### Data Analysis

Relationships between variables were firstly analyzed using correlation. To assess the unique predictive value of inattention on the two verbal WM tasks as per Diamond’s hypothesis, hierarchical regressions were carried out. For both, WM scores served as the criterion, and ADHD symptoms (along with IQ for control purposes) were added as the predictors. This was to enable us to look at the variance accounted for by inattention when hyperactivity and impulsivity were already in the model rather than to imply any direction in the relationship.

Bayes Factors (*B*) were used to assess the strength of evidence in support of hypotheses where the *p*-value indicated no significant result. These were calculated using the procedures outlined in Dienes (2014). Proposed cut-offs for acceptance of a hypothesis (Jeffreys, 1998), states a *B* above 3 as providing substantial support for the alternative hypothesis, whilst below 1/3 provides substantial support for the null hypothesis. A *B* that falls between 1/3 and 3 deems the data insensitive as to whether the alternative or null hypothesis should be accepted. We modeled the predictions of the theory of an absence of evidence for a relationship with a half-normal whose mean and standard deviation values were for the variable inattention in the backward digit span analysis. *B*_H(0,X)_ refers to the Bayes Factors testing each hypothesis, where ‘H’ indicates a half-normal distribution, and ‘X’ the predicted standard deviation of this half-normal, against a null hypothesis of no difference.

### Results

On the CAARS questionnaire 20% of participants scored above average on the composite subscale for ADHD. For individual symptoms, 32.63% of participants scored above average for inattention, 15.79% scored above average for hyperactivity, and 12.63% scored above average for impulsivity, suggesting the most prevalent symptom for this sample was inattention. CAARS scores were normally distributed and we observed not major outliers. Means, standard deviations, and ranges for each subscale are presented in **Table 1**.

**Table 1:** Mean scores, standard deviation, and range for each CAARS:S:S subscale.

	Mean	*SD*	Range
Inattention	54.07	10.68	35–77
Hyperactivity	49.67	10.61	29–78
Impulsivity	47.85	8.65	34–74
ADHD Index	51.44	10.53	31–80

Means, standard deviations, and ranges for each WM test are presented in **Table 2**. Correlations (see **Table 3**) showed fluid intelligence (Gf) score was related to performance on backward digit span score (*p* = 0.007*)*, and Corsi blocks score (*p* < 0.001), but not OSPAN score (*p* = 0.193). Neither aspect of IQ was related to any of the ADHD subscales (*ps* > 0.05). The accuracy scores on the OSPAN task were positively correlated with the average scores for memory on the same task (*r* = 0.316*, p* = 0.002), which is typical for this task. Scores on the two verbal WM tasks were positively correlated with each other (*p* < 0.001), but neither was correlated with performance on the Corsi blocks task (*ps* > 0.05).

**Table 2:** Mean scores, standard deviation, and range for each WM test.

	Mean	*SD*	Range
Backward Digit Span	8.83	2.23	5–14
Operation Span	83.59	10.64	52.13–98.97
Corsi Blocks	9.02	1.90	4–14

**Table 3:** Correlations between IQ, CAARS subsets, and working memory scores.

			Pearson correlations							
	Mean	*SD*	1	2	3	4	5	6	7	8
(1) IQ Gc	51.79	7.69	–							
(2) IQ Gf	47.77	8.83	0.131	–						
(3) Inattention	53.85	11.26	0.142	0.139	–					
(4) Hyperactivity	49.38	11.31	0.031	0.092	0.653^∗∗^	–				
(5) Impulsivity	47.55	9.38	0.057	-0.070	0.646^∗∗^	0.737^∗∗^	–			
(6) Digit Span	8.83	2.23	0.081	0.251^∗^	-0.329^∗∗^	-0.101	-0.204^∗^	–		
(7) Ospan	83.59	10.64	0.050	-0.090	-0.217^∗∗^	-0.090	-0.120	0.379^∗∗^	–	
(8) Corsi Blocks	9.02	1.90	-0.006	0.491^∗∗^	-0.087	-0.059	-0.204^∗^	0.121	0.044	–

*^∗^p < 0.05, ^∗∗^p < 0.01.*

A significant negative relationship was found between scores for inattention and performance on the two tasks assessing verbal WM; backward digit span (*p* = 0.001), and OSPAN (*p* = 0.017), but not for the spatial span task. Contrary to predictions, significant negative relationships were also found between scores for impulsivity, and performance on the backward digit span (*p* = 0.024), and Corsi blocks (*p* = 0.024) tasks. No relationships were found between hyperactivity and any of the WM tasks (*ps* > 0.05).

As expected, IQ was a significant contributor to variance in backward digit span score (*p* = 0.028, see **Table 4**). Bayes values suggested that hyperactivity made no contribution to the model (*p* = 0.225, *B*_H(0,0.25)_ = 0.28), while data for impulsivity were insensitive (*p* = 0.104, *B*_H(0,0.25)_ = 1.03). Only inattention explained a significant amount of variance in backward digit span scores when all other variables were accounted for (*p* < 0.001).

**Table 4:** Summary of regression for IQ, hyperactivity, impulsivity, and inattention on Backward Digit Span scores.

	Variable	*b*	SE*b*	β	*t*	*R^2^*	*R^2^* change	Semi-partial correlation
Step 1						0.051	0.051^∗^	
	IQ	0.044	0.020	0.225	2.226ˆ*			
Step 2						0.066	0.015	
	IQ	0.047	0.020	0.237	2.337ˆ*			0.235
	Hyperactivity	-0.024	0.020	-0.124	-1.222			-0.123
Step 3						0.093	0.027	
	IQ	0.043	0.020	0.216	2.139ˆ*			0.214
	Hyperactivity	0.012	0.030	0.058	0.391			0.039
	Impulsivity	-0.058	0.035	-0.244	-1.641			-0.164
Step 4						0.218	0.125^∗∗^	
	IQ	0.058	0.019	0.293	3.036ˆ*			0.283
	Hyperactivity	0.047	0.029	0.237	1.606			0.150
	Impulsivity	-0.012	0.035	-0.049	-0.328			-0.031
	Inattention	-0.100	0.026	-0.505	-3.793ˆ*			-0.354

*^∗^p < 0.05, ^∗∗^p < 0.001.*

Analysis for the OSPAN task was conducted in the same way (see **Table 5**). In contrast to the findings for backward digit span, analysis suggested that data for IQ were insensitive [*F*(1,93) = 0.069, *p* = 0.793, *R*^2^ = 0.001, *B*_H(0,0.25)_ = 0.47], as was the case for the ADHD symptoms hyperactivity and impulsivity (*p* = 0.401, *B*_H(0,0.25)_ = 0.86; *p* = 0.427, *B*_H(0,0.25)_ = 1.28, respectively). The inclusion of inattention did improve the model, although *p* is just shy of significance. However, the *B* suggests that there is evidence for the alternative hypothesis (*p* = 0.064, *B*_H(0,0.25)_ = 4.66), and so it is interpreted as significant.

**Table 5:** Summary of regression for IQ, hyperactivity, impulsivity, and inattention on Ospan.

	Variable	*b*	SE*b*	β	*t*	*R^2^*	*R^2^* change	Semi-partial correlation
Step 1						0.001	0.001	
	IQ	-0.026	0.098	-0.027	-0.263			
Step 2						0.008	0.008	
	IQ	-0.018	0.098	-0.019	-0.844			-0.019
	Hyperactivity	-0.083	0.098	-0.088	-1.222			-0.088
Step 3						0.015	0.007	
	IQ	-0.028	0.099	-0.029	-0.279			-0.029
	Hyperactivity	0.004	0.147	0.004	0.028			0.003
	Impulsivity	-0.141	0.176	-0.124	-0.799			-0.083
Step 4						0.052	0.037	
	IQ	0.012	0.100	0.012	0.117			0.012
	Hyperactivity	0.095	0.153	0.101	0.624			0.064
	Impulsivity	-0.020	0.185	-0.017	-0.106			-0.011
	Inattention	-0.259	0.138	-0.275	-1.875ˆ*			-0.192

*^∗^B = sub-stantial support for hypothesis.*

Although, correlations suggested inattention might not be a good predictor of Corsi blocks performance (and that perhaps impulsivity might be), regression was carried out in the same manner for this WM task (see **Table 6**). This enabled us to make direct comparisons regarding the variables across all three WM tasks. IQ was a good predictor (*p* < 0.001), as was impulsivity (*p* = 0.045). Analysis suggested that hyperactivity made no contribution to variance on this task (*p* = 0.337, *B*_H(0,0.25)_ = 0.17). The data for inattention were insensitive (*p* = 0.337, *B*_H(0,0.25)_ = 0.39).

**Table 6:** Summary of regression for IQ, hyperactivity, impulsivity, and inattention on Corsi Blocks.

	Variable	*b*	SE*b*	β	*t*	*R^2^*	*R^2^* change	Semi-partial correlation
Step 1						0.129	0.129^∗∗^	
	IQ	0.060	0.016	0.359	3.708ˆ**			
Step 2						0.138	0.009	
	IQ	0.062	0.016	0.368	3.781ˆ**			0.366
	Hyperactivity	-0.016	0.016	-0.094	-0.966			-0.094
Step 3						0.175	0.038^∗^	
	IQ	0.058	0.016	0.344	3.566ˆ**			0.340
	Hyperactivity	0.020	0.024	0.121	0.851			0.081
	Impulsivity	-0.058	0.029	-0.289	-2.035ˆ*			-0.194
Step 4						0.179	0.004	
	IQ	0.060	0.107	0.357	3.605ˆ**			0.344
	Hyperactivity	0.025	0.025	0.151	1.001			0.096
	Impulsivity	-0.052	0.031	-0.256	-1.685			-0.161
	Inattention	-0.014	0.023	-0.085	-0.620			-0.059

*^∗^p < 0.05, ^∗∗^p < 0.001.*

### Discussion

The aim of this experiment was to test the hypothesis that symptoms of inattention, but not hyperactivity-impulsivity, are associated with lower performance on verbal WM measures, particularly a complex-span task, and that this relationship is evident in a community (non-clinical) sample. Findings provide partial support for this hypothesis; inattention predicted unique variance in performance on two measures of verbal WM; the backward digit span task, and the OSPAN task. However, only the latter is a complex span task indicating that the secondary processing element is not likely to be a factor determining this relationship. Findings regarding hyperactivity and verbal WM were mixed. For the BDS task, there is evidence to suggest hyperactivity makes no contribution to performance, however, we cannot be confident of the same for the OSPAN task as the Bayes value suggests the data were insensitive. We are also unable to draw conclusions regarding impulsivity and verbal WM as Bayes values fell within the insensitive range for both tasks. Conversely, impulsivity significantly predicted performance on the Corsi blocks task, but we are unable to draw conclusions regarding inattention for this task as the Bayes value fell within the insensitive range. However, analysis suggests we can have confidence that hyperactivity does not contribute to performance on Corsi blocks. Since relationships were identified between the WM tasks and at least one ADHD symptom we can be sure there is sufficient power to detect such relationships. Overall, our data permit us to conclude that: (1) inattention is related to verbal WM; (2) impulsivity is related to spatial WM; (3) hyperactivity is not as likely to be related to the WM tasks employed in the present study.

The findings converge with previous work that has demonstrated a relationship between inattention, and performance on the backward digit span task in child (Lui and Tannock, 2007), and adult (Kim, 2004) general population samples. However, our findings do not support the idea that *complex-span* tasks are any better at elucidating the cognitive problems experienced in PIT than simple-span tasks, as performance on both the OSPAN and BDS tasks was predicted by inattention. This finding contributes to the debate as to whether the secondary element must present new stimuli to be processed (as in OSPAN), or whether mental transformation of the target memory items (as in BDS) is enough to constitute a WM task.

There is no doubt that the backward digit span task is more difficult than the forward version which requires only serial repetition of the numbers presented, but equally it is reasonable to consider the OSPAN task to be more difficult than the BDS task, not only because of its dual-processing element, but because of the mathematical demands the secondary part of the task poses. Research by Engle et al. (1999) suggests that an interference component is a necessary element to a WM task. Using factor analysis they found the backward span task grouped with short-term memory, rather than complex-span WM tasks, suggesting that mental transformation of target information is not enough. However, Oberauer et al. (2000) found no distinction between tasks involving simple transformation, and those falling into the complex span category. Further exploration suggested the ability to resist interference, or coordinate dual streams of information in complex-span tasks, does not necessarily reflect WM capacity (Oberauer et al., 2004). Our data suggest that in terms of inattention, a simple-span task is sufficiently demanding. This is even more important when considering that the present sample were not from a population with clinical diagnoses.

Diamond (2005) suggests that verbal presentation of material to children with PIT should be avoided, as it places particularly high demand on WM. She also suggests that PIT is often associated with superior spatial skills in a trade-off with linguistic skills, although we are not aware of any empirical evidence for this. Some take the view that there is no distinction between the processes used in verbal and spatial WM (Jones et al., 1995; Kane et al., 2004; Conway et al., 2005). As previously mentioned, findings relating to ADHD and spatial WM are mixed. Studies making use of the Corsi Blocks task as a spatial WM measure with child participants have found no relationship between performance and inattention (Scheres et al., 2004; Geurts et al., 2005). However, it is worth noting that while these studies may have had non-significant *p*-values, without tests for the strength of evidence for the null (e.g., Bayes), a conclusion that there is no relationship is premature. Other research has observed a relationship between combined type or unspecified ADHD and spatial WM using the Corsi Blocks and similar tasks (McInnes et al., 2003; Westerberg et al., 2004; Sowerby et al., 2011). Unfortunately, findings from the present study are not able to contribute to this debate with regard to inattention, as we cannot confidently comment on its role. However, our results do suggest that impulsivity is moderately correlated with Corsi blocks performance, and that it is a significant predictor of performance. Impulsivity is therefore a likely contributor, and potentially sole generator, of the relationship between ADHD and spatial WM reported in the literature.

Conclusions drawn from the present data should be considered in light of some potential methodological issues. Firstly, it should be noted that the Corsi blocks task is not always regarded as a measure of WM. Since the task has no concurrent processing demands, it is regularly used as a measure of simple storage. This might be responsible for the inconclusive results found in the present work. The task was used to assess spatial WM in this study on the grounds that when the sequence to be recalled becomes longer than three or four items, so that memory load increases, central executive resources are called upon (Vandierendonck et al., 2004). Also, along with similar tasks, it has been widely used as a spatial WM measure in research on ADHD symptoms (see above). However, we concede that there may be better measures of SWM, and that these might be more sensitive to variation.

Secondly, we note that participants in the current study found the mathematical component of the OSPAN task very difficult; more so than is usually observed in this task. Turner and Engle (1989) propose that the difficulty of the secondary task needs to be demanding enough to engage WM processes and reveal individual differences in task performance, but not be so difficult as to produce floor effects. Conway et al. (2005) recommend discarding data from participants who score less than 85% accuracy on the processing component of a task; accuracy is expected to be near ceiling. However, in the current study over 80% of participants failed to meet this criterion. The 5-s time limit to solve and answer the problem and read the word aloud should not have been an issue, as this was based on the average time to complete these operations found by Unsworth et al. (2005) in a sample of 296 students. The complexity of the equations themselves was no more difficult than standard versions of the task. Additionally, distribution of IQ scores in our sample was normal. Therefore, we can only speculate as to why this was, and say that further work on complex-span tasks and inattention is needed.

## Experiment 2

The present study has so far provided support for the hypothesis that deficits in verbal WM are associated with inattention. The work also supports the idea that deficits associated with ADHD continue into adulthood, and that there are implications for un-diagnosed symptoms (regardless of DSM threshold) in the general population. Experiment 2 makes use of a novel task that in contrast to traditional measures of WM, focuses on the capacity to integrate aspects of instructions and avoid goal neglect. If as Diamond (2005) has suggested, inattention leads to difficulty with verbally presented material, it may not just be the verbal nature of the material that is relevant but the fact that instructions comprise various components that need to be integrated. The letter-monitoring task described below requires the utilization of stored information and relies on representation integrity to guide behavior. However, it is thought to tap a different form of attention than that assessed in traditional WM tasks (Duncan et al., 2008).

Duncan et al. (1996) describe a type of performance failure they call *goal neglect.* In goal neglect, participants are able to state a given rule, and yet behaviorally make no attempt to adhere to it. They developed a task sensitive to this failure, originally for use with frontal lobe damaged patients, but found the effect was also demonstrated in a normal population sample. On each trial of the task, a series of number-letter pairs are shown in quick succession in the center of a computer screen. Participants are instructed to watch the characters on either the left or right side, and to read aloud the letters, but not the numbers they see on that side. An initial cue at the beginning of each trial directs participants to which side to read from; either “WATCH LEFT” or “WATCH RIGHT,” is written in the center of the screen. Then, immediately preceding the last three character pairs, a second cue directs them to which side to watch. The second cue is either a + or - symbol flashed in the center of the screen. Irrespective of what side the participant started watching, + indicates watch right, and - indicates watch left. This determines whether the participant continues reading letters on the side they started on, or switches to reading letters on the opposite side. The +/- cue is often neglected, despite the fact it is not forgotten; participants are asked during and at the end of the task to relay the +/- rule in order to confirm understanding of it. Duncan et al. (1996) concluded that while participants were perfectly capable of obeying the rule, they were simply not doing so. They suggest that information competition is a key factor in neglect, i.e., because the switch rule comes chronologically later than other task relevant information, the quality of its representation in WM is poorer. The rule would be more likely followed if other task demands were not present, and so whilst the rule is represented it is not fully integrated into what Duncan et al. (2008) refer to as the *Task Model*. This is quite different to the secondary interference posed in complex-span tasks of WM, which is designed to increase demand on resources. Duncan et al. (2008) conducted a series of experiments to determine what kind of attention or WM limit underlies goal neglect, and found that level of neglect is determined *not* by processing demands during task execution, but by total complexity of the facts, rules, and requirements in the task model. They suggest a WM limit in constructing and maintaining the task model and that this underlies goal neglect. This WM limitation is different to those tested in traditional span tasks in several ways. Firstly, Duncan et al. (2008) argue that the quantity of information needed to be held in active storage for their task creates much greater demands on capacity than do the few items on typical span task lists. Secondly, in traditional span tasks, strings of information (digits, words, etc.) are continually discarded and updated as the task goes on meaning it is not necessary to hold them in active storage for very long. The task model, however, must be kept stable throughout the course of the task, and be ready to respond to appropriate triggers. Duncan et al. (2008) suggest that this is reflective of Baddeley’s (2000) episodic buffer. Baddeley describes this as a limited-capacity temporary-storage system that is capable of integrating information from a variety of sources. Like the slave systems, it is controlled by the central executive, which is able to retrieve information from it for the purposes of reflection, manipulation and modification. The attention of the central executive is directed consciously, meaning that it influences the content of the buffer by determining what sources of information are in focus. Duncan et al. (2008) are not the only ones to make a connection between use of instructions and WM. Other work has shown a clear link between instruction guided behavior and WM, albeit in children (Jaroslawska et al., 2016; Yang et al., 2016).

If goal neglect errors arise from attention control failures, this suggests the task described by Duncan et al. (1996) will be a good measure of executive control. We would therefore expect performance on tasks assessing goal neglect to be related to inattention, but not hyperactivity or impulsivity. The existing literature on this is limited. There is evidence for an association between ADHD and goal neglect (Shue and Douglas, 1992; Pennington and Ozonoff, 1996; Karatekin, 2006; Kofman et al., 2008; van Lambalgen et al., 2008), and findings from a study using tasks designed to tap the episodic buffer suggested that ADHD was associated with an impaired ability to utilize the information processed by the buffer (Alderson et al., 2014). In terms of inattention specifically, Whyte et al. (2000) found patients with traumatic brain injury associated with impairments of attention, demonstrated increased off-task behavior, which they suggested could reflect a reduction in task-goal maintenance.

A second literature links goal neglect to *mind wandering* (McVay and Kane, 2009; Kane and McVay, 2012); the tendency to be distracted by thoughts unrelated to the task at hand. Mind wandering is also associated with ADHD (Shaw and Giambra, 1993; McVay et al., 2008), including non-clinical symptomology (Seli et al., 2015), and is reminiscent of the distraction and lack of sustained attention to tasks described in DSM criteria for inattention.

In order to extend previous findings, the present experiment aims to test whether this novel assessment of construction of WM representations, is predicted by inattention in a non-clinical sample of adults. Again, whilst not directly predicted by Diamond (2005), the notion strongly chimes with her contention that verbally presented material is particularly problematic for those with inattention, although it is not the verbal nature of the instructions that is key under present predictions, but the requirement to fully integrate and to sufficiently maintain different components of a task model. We predicted that goal neglect errors will increase with higher levels of inattention, but not be related to impulsivity or hyperactivity.

### Method

#### Participants

The 95 participants from the previous study were invited back to complete an additional WM measure; 66 accepted (men: *N* = 24, women: *N* = 42, mean age = 22.09, *SD* = 4.73).

### Materials

#### Letter Monitoring Task (Duncan et al., 1996)

**FIGURE 1:**
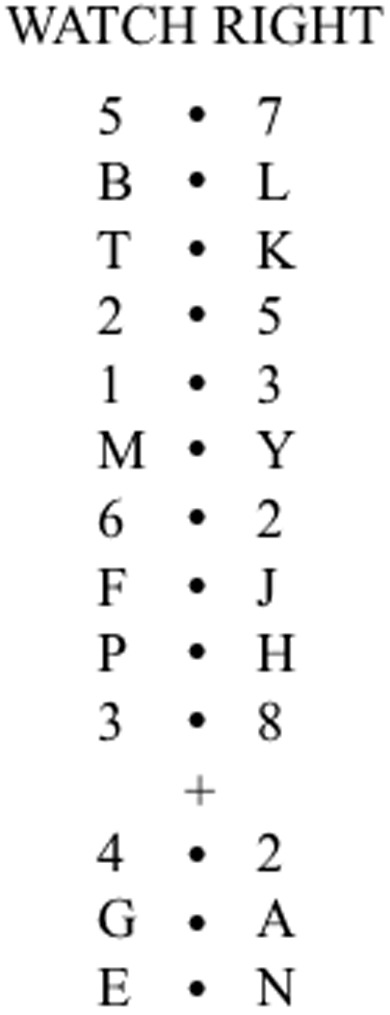
**An example of stimuli for one trial as per Duncan et al. (1996).** Time runs from top to bottom. “WATCH RIGHT” is presented for 1 s, each proceeding stimulus is presented for 200 ms separated by a blank interval of 200 ms.

The task was administered as per Duncan et al. (1996). Each block began with the word “READY” presented in the center of the screen. After the participant confirmed they were ready to proceed, the experimenter pressed a key and the word disappeared and was followed by a blank interval of 500 ms before the instruction “WATCH LEFT/RIGHT” was presented in its place for 1 s. After a further blank interval of 1 s the stimuli sequence began. This was a series of stimulus pairs (numbers or letters) presented for 200 ms and separated from the next by blank intervals of 200 ms. Ten pairs appeared in turn and after the 10th a + or - symbol was presented in the center of the screen for 200 ms. After a further blank interval of 200 ms three more pairs were presented. Of the first 10 pairs a randomly selected five were letter pairs, and the rest were numbers. Of the last three pairs, the first were always digits, and the second two were letters. On half the trials participants were to stay on the side of the initial instruction, and on the other half they were required to switch to the opposite side. For each trial digits were selected randomly and independently from the set 1–8, and letters were selected randomly without replacement from the alphabet but excluding D, I, O, V, and W. For each trial a perfectly correct report would consist of five letters from the appropriate side from the first 10 stimulus pairs, and two from the appropriate side for the last three. A prepared sheet was use to record responses for later scoring. **Figure 1** shows an example of stimuli for one trial.

Each participant received the same instructions which described the three basic task requirements: (a) to read aloud letters and ignore numbers, (b) to begin on the side as instructed on screen, (c) and to use the +/- symbols to guide responses for the final part of each trial. To ensure that the +/- rule would be remembered, pieces of paper were placed on the appropriate sides of the testing desk with either “PLUS” or “MINUS” written on them.

Participants were given at least one practice trial. Practice was repeated until at least one letter was reported (not necessarily from the correct side), and the +/- rule was described correctly.

The experiment consisted of three blocks of 12 trials. Each block contained four sub-blocks arranged so that one of each trial type (WATCH RIGHT followed by +, WATCH RIGHT followed by -, WATCH LEFT followed by -and WATCH LEFT followed by +) appeared once per sub-block in random order (equating to two “switch” trials, and two “stay” trials per sub-block). Participants were asked to repeat the rule again between each block. Verbal prompts from the experimenter were controlled in the same manor as in Duncan et al. (1996).

Scores were awarded for the number of passed sub-blocks, meaning out of the four trials within each sub-block, participants had to correctly respond to one “switch” trial, and one “stay” trial to score a point. A correct trial response amounted to correctly reading at least three pre-+/- cue letters, and at least one post- +/- cue letter.

### Results

The mean score on the letter-monitoring task was 7.02 (*SD* = 2.50, range = 1–9). Correlations (see **Table 7**) showed that, neither aspect of IQ was related to performance on the letter monitoring task (*ps* > 0.05). Performances on the three traditional tests of WM (backward digit span, OSPAN, and Corsi blocks) were all significantly positively correlated with performance on the letter monitoring task (*ps* < 0.01). As expected, the ADHD symptom inattention was significantly negatively correlated with letter monitoring performance (*p* = 0.032), while there was no significant relationship between hyperactivity or impulsivity and the task (*ps* > 0.05).

**Table 7:** Correlations between IQ, CAARS subsets, and working memory scores.

			Pearson Correlations							
	Mean	*SD*	1	2	3	4	5	6	7	8	9
(1) IQ gC	51.86	0.05	–								
(2) IQ gF	48.67	9.47	0.139	–							
(3) Inattention	54.58	10.81	0.006	0.126	–						
(4) Hyperactivity	50.27	10.73	-0.109	0.080	0.567^∗∗^	–					
(5) Impulsivity	47.97	8.90	-0.048	-0.039	0.597^∗∗^	0.658^∗∗^	–				
(6) Digit Span	8.77	2.13	0.208	0.306^∗∗^	-0.411^∗∗^	-0.140	-0.257^∗^	–			
(7) Ospan	83.45	9.63	0.091	-0.150	-0.344^∗∗^	-0.081	-0.182	0.305^∗∗^	–		
(8) Corsi Blocks	9.12	1.86	0.084	0.593^∗∗^	-0.089	-0.040	-0.184	0.194	0.124	–	
(9) Letter-Monitoring	7.02	2.50	0.152	0.170	-0.229^∗^	0.057	-0.017	0.296^∗∗^	0.352^∗∗^	0.313^∗∗^	–

*^∗^p < 0.05, ^∗∗^p < 0.01.*

Regression was used in the same manner as in Experiment 1, to assess the predictive value of inattention on letter monitoring task performance when the other symptoms of ADHD were accounted for (see **Table 8**). As before, IQ was included as a covariate along with hyperactivity and impulsivity. Bayes Factors were used as per Experiment 1. Results suggest that data for IQ were insensitive (*p* = 0.126, *B*_H(0,0.25)_ = 0.61), but that neither hyperactivity nor impulsivity contributed to variance in letter-monitoring scores (*p* = 0.431, *B*_H(0,0.25)_ = 0.16; *p* = 0.968, *B*_H(0,0.25)_ = 0.27, respectively). Inattention explained a significant amount of unique variance even after all other variables had been accounted for (*p* = 0.005).

**Table 8:** Summary of regression for IQ, hyperactivity, impulsivity, and inattention on Letter-Monitoring scores.

	Variable	*b*	SE*b*	β	*t*	*R^2^*	*R^2^* change	Semi-partial correlation
Step 1						0.036	0.036	
	IQ	0.039	0.025	0.190	1.551			0.190
Step 2						0.040	0.003	
	IQ	0.039	0.026	0.190	1.542			0.190
	Hyperactivity	0.013	0.029	0.057	0.464			0.057
Step 3						0.043	0.003	
	IQ	0.038	0.026	0.186	1.490			0.185
	Hyperactivity	0.025	0.038	0.108	0.654			0.081
	Impulsivity	-0.022	0.046	-0.077	-0.466			-0.058
Step 4						0.158	0.116^∗^	
	IQ	0.048	0.025	0.232	1.950			0.229
	Hyperactivity	0.056	0.038	0.242	1.485			0.174
	Impulsivity	0.029	0.047	0.104	0.620			0.073
	Inattention	-0.103	0.036	-0.447	-2.894^∗^			-0.340

*^∗^p < 0.01.*

### Discussion

The aim of Experiment 2 was to build on the findings of Experiment 1, and show that symptoms of inattention but not hyperactivity or impulsivity are related to WM using a novel paradigm quite different to that used in traditional tests. Our findings support this; performance on the letter-monitoring task was predicted by inattention, but not by other symptoms of ADHD. This is the first study to show that inattention is a good predictor of goal neglect.

This has implications for our understanding of the way in which WM is deficient in inattention. Our findings suggest that inattention relates to the ability to integrate a task model thought to reflect the episodic buffer component of WM. While there appeared to be no problems in construction of this knowledge base (all participants were able to repeat task rules between blocks), it seems that in inattention, the quality of it was not sufficient in order to utilize the information while concurrently undertaking the task itself. This supports previous work linking attention problems to the episodic buffer (Alderson et al., 2014) and difficulties with task-goal maintenance (Whyte et al., 2000). We suggest that this apparent difficulty with task model integration may explain why verbal presentation of information is particularly problematic for those with inattention, as highlighted by Diamond (2005).

Research has shown that construction and maintenance of the task model is strongly related to fluid intelligence (Duncan et al., 1996, 2008, 2012). The present work did not find a significant correlation between either aspect of IQ and performance on the letter-monitoring task. However, Bayes values for the regression were insensitive meaning that our data does not rule out a relationship. Although our distribution for IQ was normal, we note that Duncan et al. have often sampled from both young and old populations to ensure enough variability in IQ in their samples, which could account for our inconclusive results. Notably, our results show that inattention predicts goal neglect independently of IQ and as such our results have implications for understanding goal neglect more generally.

## General Discussion

The present research presents several important insights regarding the WM problems associated with inattention. Neither the mode (visual vs. spatial) nor the format (simple vs. complex) of presentation appears to be the factor determining the relationship between WM and inattention. Of the three ADHD symptoms, we can only be confident that inattention is associated with verbal WM deficits, which is broadly supportive of Diamond’s hypothesis. However, only the goal neglect task clearly differentiated inattention from the other two core ADHD symptoms. Results for hyperactivity provided evidence for no contribution to the backward digit span and letter-monitoring verbal WM tasks, but were inconclusive for the Ospan verbal WM task. For impulsivity, the results provided evidence for no contribution to letter-monitoring, but were inconclusive for backward digit span and Ospan.

Both experiments suggest that use of a complex-span task (involving storage plus a secondary processing element) is unnecessary to show the WM deficit associated with inattention. Performance on a simple-span task was predicted by inattention in Experiment 1, and in Experiment 2 it predicted goal neglect errors, which Duncan et al. (2008) showed are not affected by processing demands during task execution. However, as noted above, only the goal neglect task clearly differentiated the three core ADHD symptoms.

Instead, construction of complex representations sufficient enough in quality to enable use of all represented information might be the key factor differentiating WM deficits associated with inattention from those associated with the other two core ADHD symptoms. This could explain why cumulatively, research has not found a distinction between verbal and spatial WM in ADHD using traditional tasks. Nevertheless, since the goal neglect task utilized in the present study was a verbal task, future research will need to be directed to investigating this possibility. Moreover, whilst task model complexity was shown to be related to inattention in Experiment 2, it cannot be the sole factor determining WM deficits in those with inattention since the BDS task does not require the construction of a complex task model. Again though, our data do not allow us to differentiate between inattention and impulsivity in predicting performance on the BDS.

Finally, we provide evidence that symptoms of inattention experienced by adults from a community sample are associated with similar cognitive deficits as those seen in the childhood literature. It also shows that there is sufficient variation in symptomology within the general population to produce these effects. If we accept a dimensional view of ADHD and it’s symptoms, our findings have relevance to understanding them in clinical groups. Awareness of the WM deficit associated with inattention even in a non-clinical sample may be of use in developing interventions for adults experiencing difficulties.

An important theoretical issue that should be noted with relation to our findings is that of direction of causation. We use regression to analyze data for both experiments with the primary purpose of looking at inattention independently from hyperactivity and impulsivity, a by-product of which is an imposition of direction on the data. However, we mean to make no judgment on whether inattention produces the problems seen in WM, or whether WM underpins inattention. Either of these directions is reasonable. Holmes et al. (2014) suggest that high levels of inattentive and distractible behavior may arise in part from a failure to maintain task goals in WM. Such a view proposes that inattention is the behavior, i.e., the final consequence that results from a cognitive deficit pathway. This seems to be the most common view in the literature, but then much of the research in the field of ADHD is pre-geared toward developing causal models for the disorder. Alternatively, it is possible that inattention leads to poor WM, as by its very nature it limits the information, or quality of information coming into the system through lack of focus. It is also likely that there are multiple pathways to poor WM. In this work, inattention only explained a small portion of impaired WM performance. There might be another impaired process influencing performance. For example, Hofmann et al. (2012) recently proposed that self-regulation (broadly defined as goal-directed behavior) is connected to all three core EFs, and is seen as facilitating WM in several ways; primarily through top–down control of attention toward goal-relevant stimuli, and away from irrelevant stimuli. It is possible that components of self-regulation may be driving the relationship between inattention and WM, and that inattention may be better explained by a self-regulatory rather than specific EF deficit. This has not been considered specifically in relation to inattention, but Shiels and Hawk (2010) suggest that ADHD may involve deficits in self-monitoring and adaptive control components of self-regulation, and the cognitive-energetic model of ADHD (Sergeant, 2000) comes closest to representing this theoretically. Both the second and third levels of the model (the energetic pools and the management/evaluation mechanism) involve self-regulatory functions that would play a role in constructing and utilizing WM representations. Shiels and Hawk suggest that such regulatory deficit models combine cognitive and motivational theories to offer a plausible alternative to core cognitive models that don’t seem to fit with the heterogeneous nature of ADHD.

### Limitations and Future Directions

Our research used self-report measures to assess inattention and the other symptoms of ADHD, but we note that it may be useful for future work to utilize objective measures. While no such measures address DSM defined inattention *per se*, there are tasks that tap various aspects of attention, for example the Attention Network Test (ANT; Fan et al., 2002), which purports to tap three networks of attention. Redick and Engle (2006) found that WM was related to the *executive control* network (involved in resolving action conflict), and conclude that the ability to control attention is influenced by individual differences in WM capacity. It would be useful to understand whether and how the ANT relates to subjective estimates of inattention.

It is worth noting that there is evidence to suggest there may be gender differences in ADHD; both in presentation and diagnosis. Historically, males have been more likely to receive a diagnosis than females, although some have blamed this on the propensity of females to have internalizing symptoms without the more blatant externalizing symptoms (Gershon and Gershon, 2002), or clinician gender bias (Bruchmüller et al., 2012). However, data collected from university students suggested that males had a greater prevalence of both inattention and hyperactivity-impulsivity (Elisa and Parris, in review). The gender split in the current research was uneven. However, it was not the aim of the work to assess gender differences, but to establish a relationship between inattention and WM. Future work may wish to take this further by looking at whether this relationship is mediated by gender and ensuring equal numbers in each group.

In summary, we have presented evidence that WM is related to ADHD symptoms in adults without a clinical diagnosis of ADHD. However, results from both experiments suggest that inattention is the key symptom implicated in WM deficits. Whilst inconclusive for spatial WM, inattention predicted performance on both simple and complex-span traditional verbal tasks, as well as a novel task assessing WM for task rules.

## Author Contributions

RE designed, implemented, and wrote up the work. EB-B and BP supervised the work, and contributed to analysis and write up.

## Conflict of Interest Statement

The authors declare that the research was conducted in the absence of any commercial or financial relationships that could be construed as a potential conflict of interest.

## References

[B1] Alderson R. M. Kasper L. J. Hudec K. L. Patros C. H. (2013). Attention-deficit/hyperactivity disorder (ADHD) and working memory in adults: a meta-analytic review. *Neuropsychology* 27 287–302. 10.1037/a0032371 23688211

[B2] Alderson R. M. Kasper L. J. Patros C. H. Hudec K. L. Tarle S. J. Lea S. E. (2014). Working memory deficits in boys with attention deficit/hyperactivity disorder (ADHD): an examination of orthographic coding and episodic buffer processes. *Child Neuropsychol.* 21 509–530. 10.1080/09297049.2014.917618 24830472

[B3] Alloway T. Elliott J. Holmes J. (2010). The prevalence of AD (H) D-Like symptoms in a community sample. *J. Atten. Disord.* 14 52–56. 10.1177/1087054709356197 20378922

[B4] American Psychiatric Association (2013). *Diagnostic and Statistical Manual of Mental Disorders*, 5th Edn. Arlington, VA: American Psychiatric Publishing.

[B5] Baddeley A. (2000). The episodic buffer: a new component of working memory? *Trends Cogn. Sci*, 4 417–423. 10.1016/S1364-6613(00)01538-2 11058819

[B6] Baddeley A. D. Hitch G. (1974). “Working Memory,” in *Psychology of Learning and Motivation: Advances in Research and Theory* Vol. 8 ed. Gordon H. B. (New York, NY: Academic Press), 47–89.

[B7] Barkley R. A. (1997). Behavioral inhibition, sustained attention, and executive functions: constructing a unifying theory of ADHD. *Psychol. Bull.* 121 65–94. 10.1037/0033-2909.121.1.65 9000892

[B8] Barkley R. A. (2001). The inattentive type of ADHD as a distinct disorder: what remains to be done. *Clin. Psychol. Sci. Pract.* 8 489–493. 10.1093/clipsy.8.4.489

[B9] Barkley R. A. Murphy K. R. (2006). *Attention-Deficit Hyperactivity Disorder: A Clinical Workbook*, Vol. 2 New York, NY: Guilford Press

[B10] Biederman J. Mick E. Faraone S. V. (2000). Age-dependent decline of symptoms of attention deficit hyperactivity disorder: impact of remission definition and symptom type. *Am. J. Psychiatry* 157 816–818. 10.1176/appi.ajp.157.5.816 10784477

[B11] Boonstra A. M. Oosterlaan J. Sergeant J. A. Buitelaar J. K. (2005). Executive functioning in adult ADHD: a meta-analytic review. *Psychol. Med.* 35 1097–1108. 10.1017/S003329170500499X 16116936

[B12] Bruchmüller K. Margraf J. Schneider S. (2012). Is ADHD diagnosed in accord with diagnostic criteria? Overdiagnosis and influence of client gender on diagnosis. *J. Consult. Clin. Psychol.* 80 128–138. 10.1037/a0026582 22201328

[B13] Chhabildas N. Pennington B. F. Willcutt E. G. (2001). A comparison of the neuropsychological profiles of the DSM-IV subtypes of ADHD. *J. Abnorm. Child Psychol.* 29 529–540. 10.1023/A:1012281226028 11761286

[B14] Conners C. K. Erhardt D. Epstein J. N. Parker J. D. A. Sitarenios G. Sparrow E. (1999). Self-ratings of ADHD symptoms in adults I: factor structure and normative data. *J. Atten. Disord.* 3 141–151. 10.1177/108705479900300303

[B15] Conway A. R. Kane M. J. Bunting M. F. Hambrick D. Z. Wilhelm O. Engle R. W. (2005). Working memory span tasks: a methodological review and user’s guide. *Psychon. Bull. Rev.* 12 769–786. 10.3758/BF03196772 16523997

[B16] Conway A. R. A. Engle R. W. (1994). Working memory and retrieval: a resource-dependent inhibition model. *J. Exp. Psychol. Gen.* 123 354–373. 10.1037/0096-3445.123.4.354 7996121

[B17] Corsi P. M. (1973). *Human Memory and the Medial Temporal Region of the Brain.* Ph.D. thesis, Department of Psychology, ProQuest Information & Learning, Ann Arbor, MI.

[B18] Diamond A. (2005). Attention-deficit disorder (attention-deficit/ hyperactivity disorder without hyperactivity): a neurobiologically and behaviorally distinct disorder from attention-deficit/hyperactivity disorder (with hyperactivity). *Dev. Psychopathol.* 17 807–825. 10.1017/s0954579405050388 16262993 PMC1474811

[B19] Dienes Z. (2014). Using Bayes to get the most out of non-significant results. *Front. Psychol.* 5:781 10.3389/fpsyg.2014.00781 PMC4114196 25120503

[B20] Dowson J. H. McLean A. Bazanis E. Toone B. Young S. Robbins T. W. (2004). Impaired spatial working memory in adults with attention-deficit/hyperactivity disorder: comparisons with performance in adults with borderline personality disorder and in control subjects. *Acta Psychiatr. Scand.* 110 45–54. 10.1111/j.1600-0447.2004.00292.x 15180779

[B21] Doyle A. E. (2005). Executive functions in attention-deficit/hyperactivity disorder. *J. Clin. Psychiatry* 67 21–26. 16961426

[B22] Duncan J. Emslie H. Williams P. Johnson R. Freer C. (1996). Intelligence and the frontal lobe: the organization of goal-directed behavior. *Cogn. Psychol.* 30 257–303. 10.1006/cogp.1996.0008 8660786

[B23] Duncan J. Parr A. Woolgar A. Thompson R. Bright P. Cox S. (2008). Goal neglect and Spearman’s g: competing parts of a complex task. *J. Exp. Psychol. Gen.* 137 131–148. 10.1037/0096-3445.137.1.131 18248133

[B24] Duncan J. Schramm M. Thompson R. Dumontheil I. (2012). Task rules, working memory, and fluid intelligence. *Psychon. Bull. Rev.* 19 864–870. 10.3758/s13423-012-0225-y 22806448 PMC3456922

[B25] Egeland J. Rund B. Sundet K. Landrø N. Asbjørnsen A. Lund A. (2003). Attention profile in schizophrenia compared with depression: differential effects of processing speed, selective attention and vigilance. *Acta Psychiatr. Scand.* 108 276–284. 10.1034/j.1600-0447.2003.00146.x 12956828

[B26] Elisa R. N. Parris B. A. (2015). The relationship between core symptoms of ADHD and the cognitive reflection test in a non-clinical sample. *Cogn. Neuropsychiatry* 20 416–423. 10.1080/13546805.2015.1068687 26288014

[B27] Elliott H. (2002). Attention deficit hyperactivity disorder in adults: a guide for the primary care physician. *South. Med. J.* 95 736–742. 10.1097/00007611-200207000-00015 12144080

[B28] Engle R. W. Tuholski S. W. Laughlin J. E. Conway A. R. (1999). Working memory, short-term memory, and general fluid intelligence: a latent-variable approach. *J. Exp. Psychol. Gen.* 128 309–331. 10.1037/0096-3445.128.3.309 10513398

[B29] Fan J. McCandliss B. D. Sommer T. Raz A. Posner M. I. (2002). Testing the efficiency and independence of attentional networks. *J. Cogn. Neurosci.* 14 340–347. 10.1162/089892902317361886 11970796

[B30] Faraone S. V. Biederman J. (2005). What is the prevalence of adult ADHD? Results of a population screen of 966 adults. *J. Atten. Disord.* 9 384–391. 16371661 10.1177/1087054705281478

[B31] Gansler D. A. Fucetola R. Krengel M. Stetson S. Zimering R. Makary C. (1998). Are there cognitive subtypes in adult attention deficit/hyperactivity disorder? *J. Nerv. Ment. Dis.* 186 776–781. 10.1097/00005053-199812000-00006 9865816

[B32] Gershon J. Gershon J. (2002). A meta-analytic review of gender differences in ADHD. *J. Atten. Disord.* 5 143–154. 10.1177/108705470200500302 11911007

[B33] Geurts H. M. Verté S. Oosterlaan J. Roeyers H. Sergeant J. A. (2005). ADHD subtypes: do they differ in their executive functioning profile? *Arch. Clin. Neuropsychol.* 20 457–477. 10.1016/j.acn.2004.11.001 15896560

[B34] Hervey A. S. Epstein J. N. Curry J. F. (2004). Neuropsychology of adults with attention-deficit/hyperactivity disorder: a meta-analytic review. *Neuropsychol.* 18 485–503. 10.1037/0894-4105.18.3.485 15291727

[B35] Hofmann W. Schmeichel B. J. Baddeley A. D. (2012). Executive functions and self-regulation. *Trends Cogn. Sci.* 16 174–180. 10.1016/j.tics.2012.01.006 22336729

[B36] Holmes J. Hilton K. A. Place M. Alloway T. P. Elliott J. G. Gathercole S. E. (2014). Children with low working memory and children with ADHD: same or different? *Front. Hum. Neurosci.* 8:976 10.3389/fnhum.2014.00976 PMC4260512 25538599

[B37] Jaroslawska A. J. Gathercole S. E. Logie M. R. Holmes J. (2016). Following instructions in a virtual school: does working memory play a role? *Mem. Cognit.* 44 580–589. 10.3758/s13421-015-0579-2 PMC4835523 26680246

[B38] Jeffreys H. (1998). *The Theory of Probability.* Oxford: Oxford University Press.

[B39] Johnson D. E. Epstein J. N. Waid L. R. Latham P. K. Voronin K. E. Anton R. F. (2001). Neuropsychological performance deficits in adults with attention deficit/hyperactivity disorder. *Arch. Clin. Neuropsychol.* 16 587–604. 10.1016/S0887-6177(00)00070-6 14590156

[B40] Jones D. Farrand P. Stuart G. Morris N. (1995). Functional equivalence of verbal and spatial information in serial short-term memory. *J. Exp. Psychol. Learn. Mem. Cogn.* 21 1008–1018. 7673864 10.1037//0278-7393.21.4.1008

[B41] Kane M. J. Engle R. W. (2000). Working-memory capacity, proactive interference, and divided attention: limits on long-term memory retrieval. *J. Exp. Psychol. Learn. Mem. Cogn.* 26 336–358. 10.1037/0278-7393.26.2.336 10764100

[B42] Kane M. J. Engle R. W. (2002). The role of prefrontal cortex in working-memory capacity, executive attention, and general fluid intelligence: an individual-differences perspective. *Psychon. Bull. Rev.* 9 637–671. 10.3758/BF03196323 12613671

[B43] Kane M. J. Hambrick D. Z. Tuholski S. W. Wilhelm O. Payne T. W. Engle R. W. (2004). The generality of working memory capacity: a latent-variable approach to verbal and visuospatial memory span and reasoning. *J. Exp. Psychol. Gen.* 133 189–217. 10.1037/0096-3445.133.2.189 15149250

[B44] Kane M. J. McVay J. C. (2012). What mind wandering reveals about executive-control abilities and failures. *Curr. Dir. Psychol. Sci.* 21 348–354. 10.1177/0963721412454875

[B45] Karatekin C. (2006). Improving antisaccade performance in adolescents with attention-deficit/hyperactivity disorder (ADHD). *Exp. Brain Res.* 174 324–341. 10.1007/s00221-006-0467-x 16639499

[B46] Kasper L. J. Alderson R. M. Hudec K. L. (2012). Moderators of working memory deficits in children with attention-deficit/hyperactivity disorder (ADHD): a meta-analytic review. *Clin. Psychol. Rev.* 32 605–617. 10.1016/j.cpr.2012.07.001 22917740

[B47] Kim S.-J. (2004). *Neurocognitive Features of Attention Deficit Hyperactivity Disorder in a Non-Clinical Adult Sample.* Master’s thesis, Uniformed Services University of the Health Sciences, Bethesda, MDQ.

[B48] Klingberg T. Fernell E. Olesen P. J. Johnson M. Gustafsson P. Dahlström K. (2005). Computerized training of working memory in children with ADHD-a randomized, controlled trial. *J. Am. Acad. Child Adolesc. Psychiatry* 44 177–186. 10.1097/00004583-200502000-00010 15689731

[B49] Kofman O. Gidley Larson J. Mostofsky S. H. (2008). A novel task for examining strategic planning: Evidence for impairment in children with ADHD. *J. Clin. Exp. Neuropsychol.* 30 261–271. 10.1080/13803390701380583 17852623

[B50] Lui M. Tannock R. (2007). Working memory and inattentive behaviour in a community sample of children. *Behav. Brain Funct.* 3:12 10.1186/1744-9081-3-12 PMC1820786 17319951

[B51] Marchetta N. D. Hurks P. P. Krabbendam L. Jolles J. (2008). Interference control, working memory, concept shifting, and verbal fluency in adults with attention-deficit/hyperactivity disorder (ADHD). *Neuropsychology* 22 74–84. 10.1037/0894-4105.22.1.74 18211157

[B52] Martinussen R. Hayden J. Hogg-Johnson S. Tannock R. (2005). A meta-analysis of working memory impairments in children with attention-deficit/hyperactivity disorder. *J. Am. Acad. Child Adolesc. Psychiatry* 44 377–384. 10.1097/01.chi.0000153228.72591.73 15782085

[B53] Martinussen R. Tannock R. (2006). Working memory impairments in children with attention-deficit hyperactivity disorder with and without comorbid language learning disorders. *J. Clin. Exp. Neuropsychol.* 28 1073–1094. 10.1080/13803390500205700 16840237

[B54] Mayes S. D. Calhoun S. L. Mayes R. D. Molitoris S. (2012). Autism and ADHD: overlapping and discriminating symptoms. *Res. Autism Spectr. Disord.* 6 277–285. 10.1016/j.rasd.2011.05.009

[B55] McInnes A. Humphries T. Hogg-Johnson S. Tannock R. (2003). Listening comprehension and working memory are impaired in attention-deficit hyperactivity disorder irrespective of language impairment. *J. Abnorm. Child Psychol.* 31 427–443. 10.1023/A:1023895602957 12831231

[B56] McVay J. Knouse L. Mitchell J. Brown L. Kane M. J. Kwapil T. R. (2008). Impaired conductors in the train of thought? Mind wandering in attention deficit/hyperactivity disorder. *Paper presented at the Poster presented at the annual meeting of Association for Psychological Science*, Chicago, IL.

[B57] McVay J. C. Kane M. J. (2009). Conducting the train of thought: working memory capacity, goal neglect, and mind wandering in an executive-control task. *J. Exp. Psychol. Learn. Mem. Cogn.* 35 196–204. 10.1037/a0014104 19210090 PMC2750806

[B58] Milich R. Balentine A. C. Lynam D. R. (2001). ADHD combined type and ADHD predominantly inattentive type are distinct and unrelated disorders. *Clin. Psychol. Sci. Pract.* 8 463–488. 10.1093/clipsy.8.4.463

[B59] Millstein R. B. Wilens T. E. Biederman J. Spencer T. J. (1997). Presenting ADHD symptoms and subtypes in clinically referred adults with ADHD. *J. Atten. Disord.* 2 159–166. 10.1177/108705479700200302

[B60] Murphy K. Barkley R. A. (1996). Prevalence of DSM-IV symptoms of ADHD in adult licensed drivers: implications for clinical diagnosis. *J. Atten. Disord.* 1 147–161. 10.1177/108705479600100303

[B61] Nigg J. T. Blaskey L. G. Huang-Pollock C. L. Rappley M. D. (2002). Neuropsychological executive functions and DSM-IV ADHD subtypes. *J. Am. Acad. Child Adolesc. Psychiatry* 41 59–66. 10.1097/00004583-200201000-00012 11800208

[B62] Oberauer K. Lange E. Engle R. W. (2004). Working memory capacity and resistance to interference. *J. Mem. Lang.* 51 80–96. 10.1016/j.jml.2004.03.003

[B63] Oberauer K. Süß H.-M. Schulze R. Wilhelm O. Wittmann W. (2000). Working memory capacity—facets of a cognitive ability construct. *Pers. Individ. Dif.* 29 1017–1045. 10.1016/S0191-8869(99)00251-2

[B64] Pennington B. F. Ozonoff S. (1996). Executive functions and developmental psychopathology. *J. Child Psychol. Psychiatry* 37 51–87. 10.1111/j.1469-7610.1996.tb01380.x 8655658

[B65] Redick T. S. Engle R. W. (2006). Working memory capacity and attention network test performance. *Appl. Cogn. Psychol.* 20 713–721. 10.1002/acp.1224

[B66] Redick T. S. Lindsey D. R. (2013). Complex span and n-back measures of working memory: a meta-analysis. *Psychon. Bull. Rev.* 20 1102–1113. 10.3758/s13423-013-0453-9 23733330

[B67] Scheres A. Oosterlaan J. Geurts H. Morein-Zamir S. Meiran N. Schut H. (2004). Executive functioning in boys with ADHD: primarily an inhibition deficit? *Arch. Clin. Neuropsychol.* 19 569–594. 10.1016/j.acn.2003.08.005 15163457

[B68] Schmitz M. Cadore L. Paczko M. Kipper L. Chaves M. Rohde L. A. (2002). Neuropsychological performance in DSM-IV ADHD subtypes: an exploratory study with untreated adolescents. *Can. J. Psychiatry* 47 863–869. 10.1177/070674370204700908 12500757

[B69] Schoechlin C. Engel R. R. (2005). Neuropsychological performance in adult attention-deficit hyperactivity disorder: meta-analysis of empirical data. *Arch. Clin. Neuropsychol.* 20 727–744. 10.1016/j.acn.2005.04.005 15953706

[B70] Schweitzer J. B. Hanford R. B. Medoff D. R. (2006). Working memory deficits in adults with ADHD: is there evidence for subtype differences. *Behav. Brain Funct.* 2 43 10.1186/1744-9081-2-43 PMC1762010 17173676

[B71] Seidman L. J. Biederman J. Weber W. Hatch M. Faraone S. V. (1998). Neuropsychological function in adults with attention-deficit hyperactivity disorder. *Biol. Psychiatry* 44 260–268. 10.1016/S0006-3223(97)00392-2 9715357

[B72] Seitz J. Kahraman-Lanzerath B. Legenbauer T. Sarrar L. Herpertz S. Salbach-Andrae H. (2013). The role of impulsivity, inattention and comorbid ADHD in patients with bulimia nervosa. *PLoS ONE* 8:e63891 10.1371/journal.pone.0063891 PMC3659086 23700439

[B73] Seli P. Smallwood J. Cheyne J. A. Smilek D. (2015). On the relation of mind wandering and ADHD symptomatology. *Psychon. Bull. Rev.* 22 629–636. 10.3758/s13423-014-0793-0 25561417

[B74] Sergeant J. (2000). The cognitive-energetic model: an empirical approach to attention-deficit hyperactivity disorder. *Neurosci. Biobehav. Rev.* 24 7–12. 10.1016/S0149-7634(99)00060-3 10654654

[B75] Shaw G. Giambra L. (1993). Task-unrelated thoughts of college students diagnosed as hyperactive in childhood. *Dev. Neuropsychol.* 9 17–30. 10.1080/87565649309540541

[B76] Shiels K. Hawk L. W. (2010). Self-regulation in ADHD: the role of error processing. *Clin. Psychol. Rev.* 30 951–961. 10.1016/j.cpr.2010.06.010 20659781 PMC2952677

[B77] Shue K. L. Douglas V. I. (1992). Attention deficit hyperactivity disorder and the frontal lobe syndrome. *Brain Cogn.* 20 104–124. 10.1016/0278-2626(92)90064-S 1389116

[B78] Sowerby P. Seal S. Tripp G. (2011). Working memory deficits in ADHD: the contribution of age, learning/language difficulties, and task parameters. *J. Atten. Disord.* 15 461–472. 10.1177/1087054710370674 20574057

[B79] Stupple E. J. N. Gale M. Richmond C. (2013). “Working memory, cognitive miserliness and logic as predictors of performance on the cognitive reflection test,” in *Proceedings of the 35th Annual Conference of the Cognitive Science Society*, eds Knauff M. Pauen M. Sebanz N. Wachsmuth I. (Austin, TX: Cognitive Science Society).

[B80] Turner M. L. Engle R. W. (1989). Is working memory capacity task dependent? *J. Mem. Lang.* 28 127–154. 10.1016/0749-596X(89)90040-5

[B81] Unsworth N. Heitz R. P. Schrock J. C. Engle R. W. (2005). An automated version of the operation span task. *Behav. Res. Methods* 37 498–505. 10.3758/BF03192720 16405146

[B82] van Lambalgen M. van Kruistum C. Parigger E. (2008). Discourse processing in attention-deficit hyperactivity disorder (ADHD). *J. Logic Lang. Inf.* 17 467–487. 10.1007/s10849-008-9066-5

[B83] Vandierendonck A. Kemps E. Fastame M. C. Szmalec A. (2004). Working memory components of the Corsi blocks task. *Br. J. Psychol.* 95 57–79. 10.1348/000712604322779460 15005868

[B84] Wender P. H. Wolf L. E. Wasserstein J. (2001). Adults with ADHD. *Ann. N. Y. Acad. Sci.* 931 1–16. 10.1111/j.1749-6632.2001.tb05770.x 11462736

[B85] Westerberg H. Hirvikoski T. Forssberg H. Klingberg T. (2004). Visuo-spatial working memory span: a sensitive measure of cognitive deficits in children with ADHD. *Child Neuropsychol.* 10 155–161. 10.1080/09297040409609806 15590494

[B86] Whyte J. Schuster K. Polansky M. Adams J. Coslett H. (2000). Frequency and duration of inattentive behavior after traumatic brain injury: effects of distraction, task, and practice. *J. Int. Neuropsychol. Soc.* 6 1–11. 10.1017/S1355617700611013 10761362

[B87] Willcutt E. G. Doyle A. E. Nigg J. T. Faraone S. V. Pennington B. F. (2005). Validity of the executive function theory of attention-deficit/hyperactivity disorder: a meta-analytic review. *Biol. Psychiatry* 57 1336–1346. 10.1016/j.biopsych.2005.02.006 15950006

[B88] Wolf L. E. Wasserstein J. (2001). Adults ADHD. *Ann. N. Y. Acad. Sci.* 931 396–408. 10.1111/j.1749-6632.2001.tb05793.x 11462756

[B89] Woods S. P. Lovejoy D. W. Ball J. (2002). Neuropsychological characteristics of adults with ADHD: a comprehensive review of initial studies. *Clin. Neuropsychol.* 16 12–34. 10.1076/clin.16.1.12.8336 11992223

[B90] Yang T. X. Allen R. J. Gathercole S. E. (2016). Examining the role of working memory resources in following spoken instructions. *J. Cogn. Psychol.* 28 186–198. 10.1080/20445911.2015.1101118

